# Supplementary figures and images for: Multi-ethnic transcriptome-wide association study of prostate cancer
Source: PLoS One. 2020 Sep 28;15(9):e0236209. doi: 10.1371/journal.pone.0236209 (PMC7521738; doi:10.1371/journal.pone.0236209)

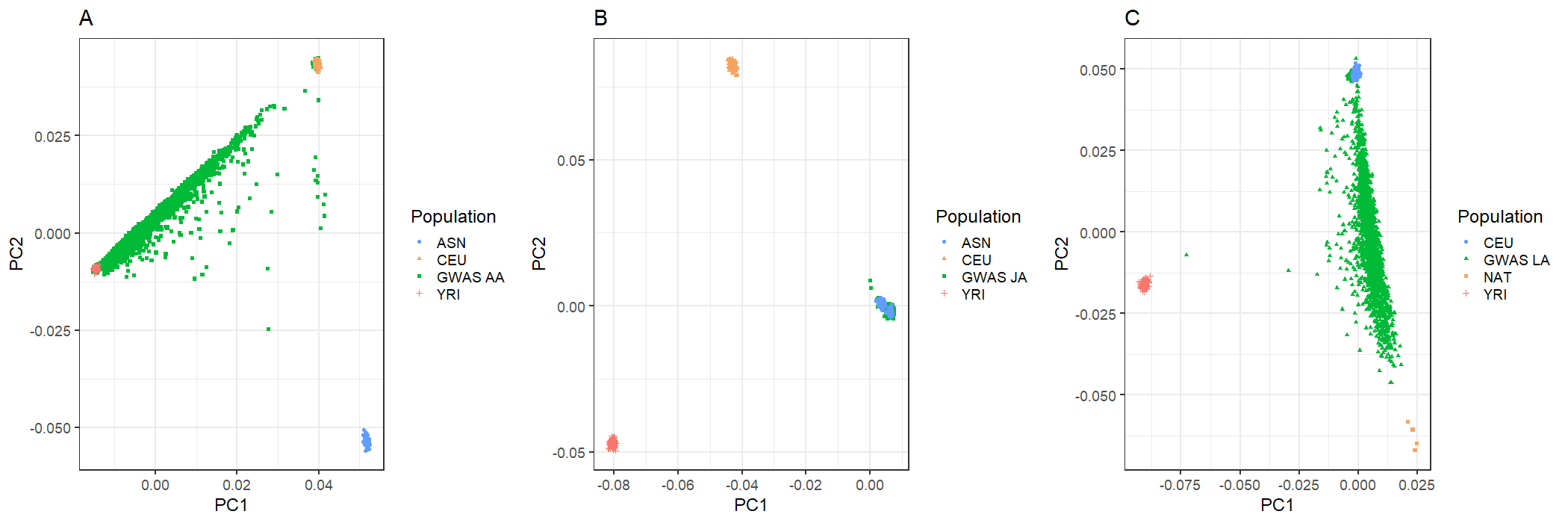

Supplement: S1 Fig — After merging genotypes with those of four reference populations from version three of the HapMap Project, we performed principal component analysis of all three study populations separately. African American (A) and Japanese American (B) genotypes are plotted with three populations from HapMap: Chinese in Beijing and Japanese in Tokyo (ASN), European ancestries in Utah (CEU), and Yoruba people in Ibadan, Nigeria (YRI). The Latin American genotypes are plotted with Chinese in Beijing and Japanese in Tokyo (ASN), European ancestries in Utah (CEU), and indigenous people of North America (NAT). (TIF) [file pone.0236209.s001.tif]

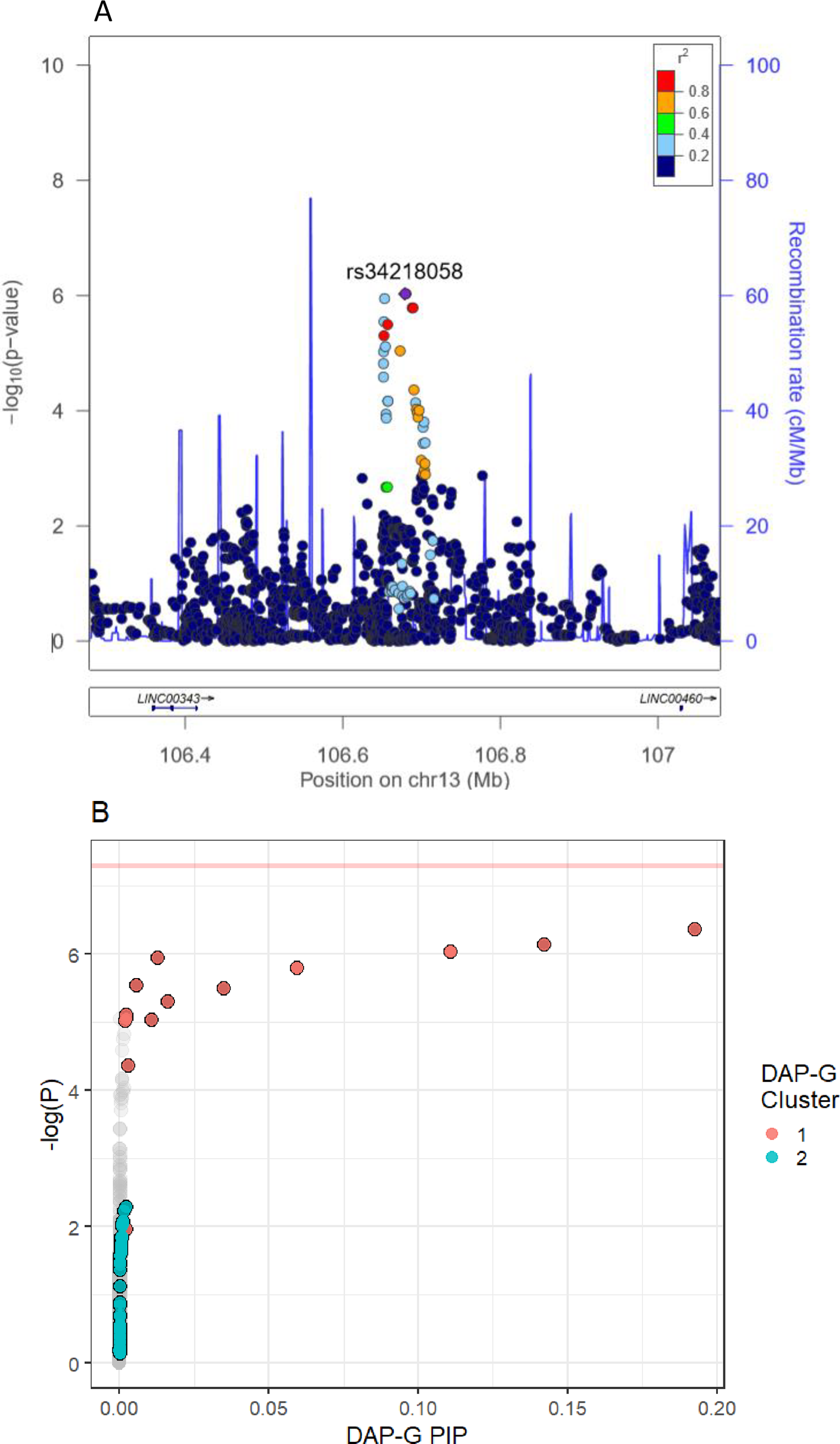

Supplement: S2 Fig — Genome-wide association studies identified no genome-wide significant SNPs. (A) depicts a LocusZoom plots of the most associated GWAS results from Native American population on chromosome 13 [35]. (A) is plotted using 1000G AMR 2014 LD. The y-axis is the -log(P) while the x-axis is location on chromosome 13 measured in megabases. Color represents the LD r2. (B) depicts the results of our GWAS in comparison to DAP-G cluster and PIP for the Latin American population [20]. Each point on the plot represents one SNP in our GWAS. The y-axis is -log(P), and the x-axis is the individual SNP PIP as calculated by DAP-G. The color of each point represents the cluster to which DAP-G assigned it. (TIF) [file pone.0236209.s002.tif]

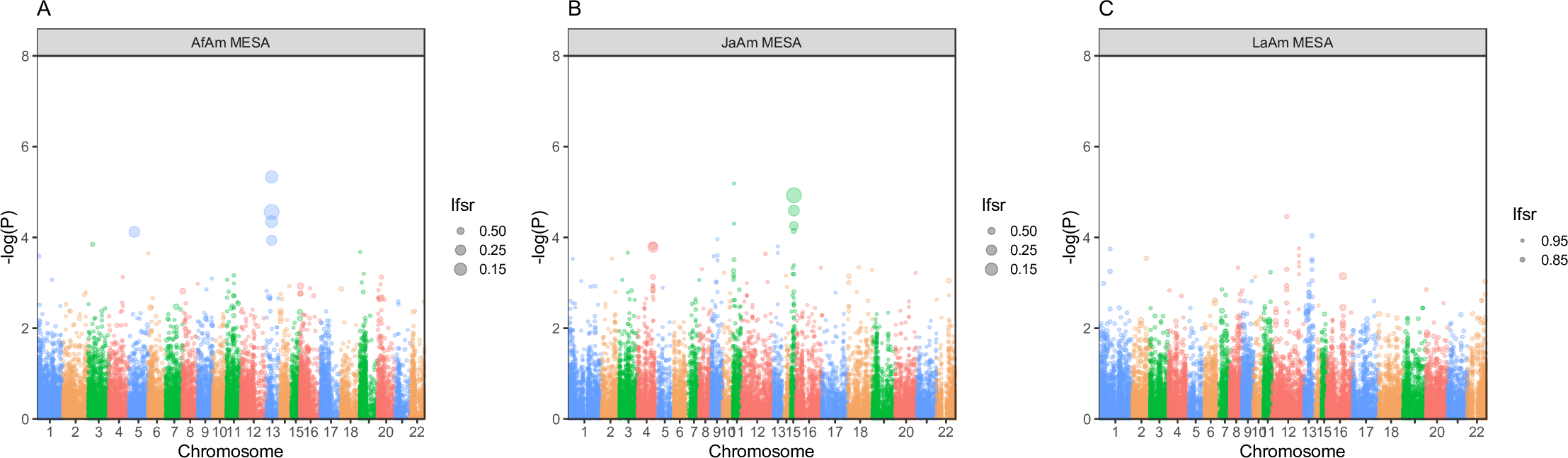

Supplement: S3 Fig — (A, B, & C) are Manhattan plots of the gene-based association study using MESA monocyte gene expression prediction models for the respective African American, Japanese American, and Latin American populations. Each point represents a gene-tissue test from PrediXcan. The y-axis represents the −log10(P) of the gene-tissue test, and the x-axis plots chromosome number. The size of the dot is inversely proportional to its lfsr. (TIF) [file pone.0236209.s003.tif]

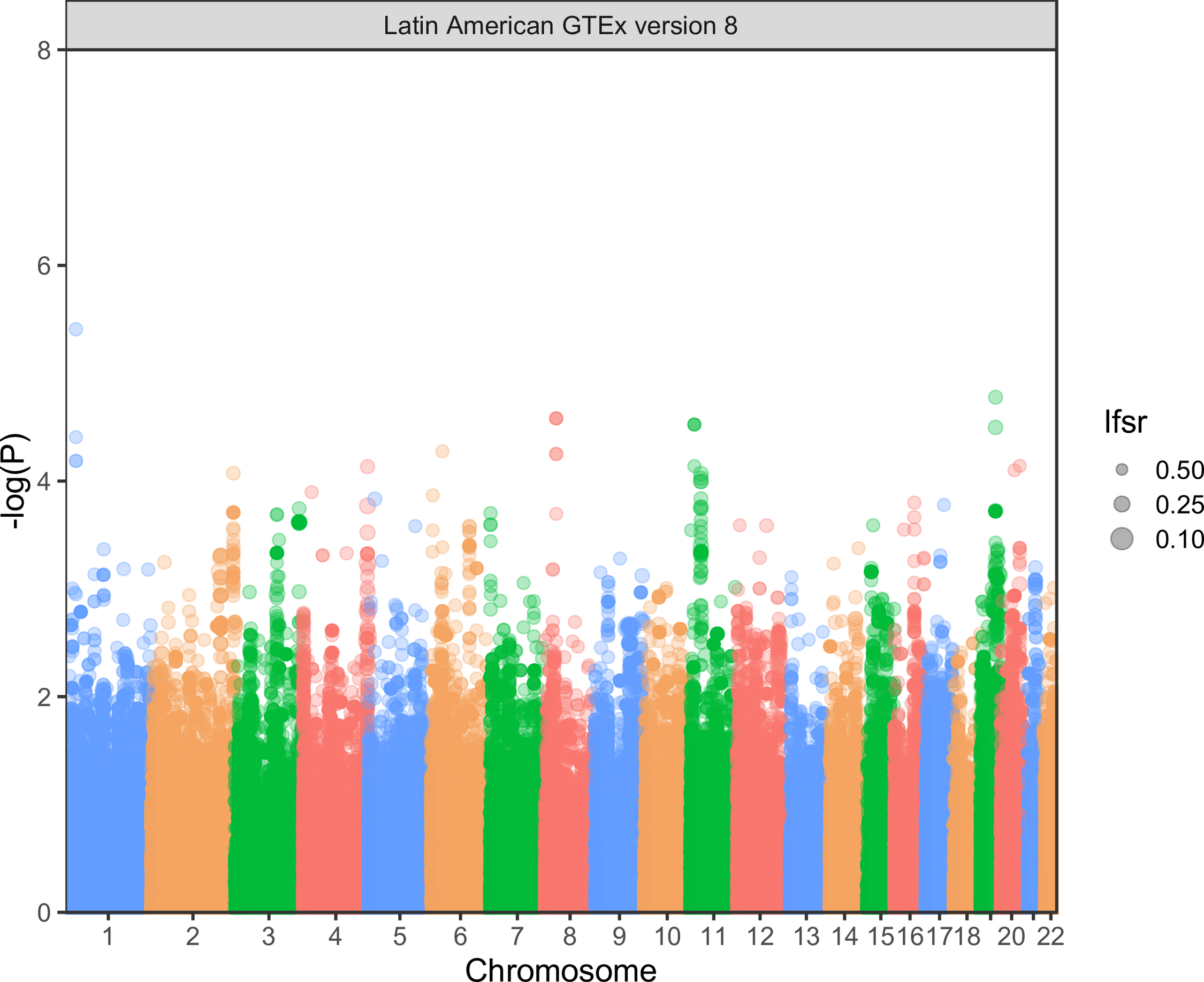

Supplement: S4 Fig — Manhattan plot of the gene-based association study using GTEx version 8 mashr gene expression prediction models for the Latin American population. Each point represents a gene-tissue test from PrediXcan. The y-axis represents the −log10(P) of the gene-tissue test, and the x-axis plots chromosome number. The size of the dot is inversely proportional to its lfsr. (TIF) [file pone.0236209.s004.tif]
